# Supplementary material for: Social context of contraceptive use transition among sexually active women in Zambia (1992–2018): A decomposition analysis
Source: PLoS One. 2024 Apr 16;19(4):e0300506. doi: 10.1371/journal.pone.0300506 (PMC11020625; doi:10.1371/journal.pone.0300506)
Supplement: Attachment — Submitted filename: Respnose to Reviewers.docx [file pone.0300506.s001.docx]

The Editor,

PLOS ONE

20^th^ January 2024

**Sub: Response to Decision Letter: Manuscript ID:** PONE-D-23-29924**:** A decomposition analysis of context of contraceptive use transition in Zambia (1992-2018)

Refer to the above subject matter.

We are grateful to the editor and reviewers for their time and constructive comments on our manuscript. We have taken into consideration the second round of rviews and suggestions the editor, and we would like to submit a revised version of the manuscript for consideration by the journal. To reflect changes or amendments made in the revised version of the manuscript, changes or amendments have been highlighted in the initial version of the manuscript. In addition, we provide a point-by-point response explaining how we have addressed the editors comments in the section following this one. We are looking forward to hearing the results of your evaluation.

Yours sincerely,

Lead author

Million Phiri

On behalf of co-authors

**Title:** A decomposition analysis of context of contraceptive use transition in Zambia (1992-2018)

| **Journal Requirements** | | |
| --- | --- | --- |
| **Editors Remarks** | **Authors Response** | **Reference** |
| Thank you for submitting your manuscript to PLOS ONE. After careful consideration, we feel that it has merit but does not fully meet PLOS ONE’s publication criteria as it currently stands. Therefore, we invite you to submit a revised version of the manuscript that addresses the points raised during the review process. | We would like to extend our gratitude to the editor and reviewers for their valuable time and constructive feedback provided on our manuscript. We have diligently worked towards addressing all the raised comments and have incorporated the suggested edits accordingly. We eagerly anticipate any additional support or guidance moving forward. |  |
| Journal Requirements:  When submitting your revision, we need you to address these additional requirements.  1. Please ensure that your manuscript meets PLOS ONE's style requirements, including those for file naming. The PLOS ONE style templates can be found at  <https://journals.plos.org/plosone/s/file?id=wjVg/PLOSOne_formatting_sample_main_body.pdf> and  <https://journals.plos.org/plosone/s/file?id=ba62/PLOSOne_formatting_sample_title_authors_affiliations.pdf> | We have ensured that all appropriate journal guideline have been followed in presenting the manuscript. | Whole manuscript document |
| **Reviewer 1** | | |
| **Questions and Comments** | **Authors Response** | **Reference** |
| The manuscript's structure is meticulously organized, allowing for a seamless flow of ideas and a coherent presentation of the research findings. However, I want to redirect the authors to the following questions and comments. | We are grateful for your favourable comments and remarks. We would like to express our appreciation for your valuable expertise and the time you dedicated to our work. |  |
| 1.) As you stated in the manuscript (Figure 3), much of the significant changes in contraceptive use occurred in the period 1992-2007, that is, a 24% increase has been recorded. Ever since 2007, the change has been sluggish and not worthy of decomposition. In my opinion, the reasons for the stall or the slower progress of contraceptive use in the recent decade (2007-2018) should be excavated and your style of analysis will neglect this fact when you emphasize only on the general rise. Why won't you focus on the decomposition of the abovementioned period 1992 to 2007? Otherwise, there is no point of using all the six DHS data for the decomposition. The first and the last would suffice. | Thank you for the guidance and the questions. We appreiciate your exepeetise.   1. The Blinder Oacaca decomposition analysis technique used to perform analysis applies a two component method. The method requires only two points to conduct a decmposition analysis. In our case we used the first (1992) and the most recent (2018) DHS to perform the decomposition analysis. The thriugh was to see what has been driving chnage bweeen the first and the last DHS. The clarifcation of the reference period for the decomposition analysis has been included in the manuscript. 2. The other four DHS were only use to describe the trend in contraceptive use but decomposition analysis was only done between the first and last DHS. 3. Similar to what been been observed in other SSA countries, the most increase in CPR happened during the earlier DHS phases. The later phases saw litte change but there is a sharp change in terms of methods used. 4. Looking forward to your further guidance | **Line 1**  **Line 88-89**  **Line 151-153** |
| 2.) As you have indicated in the manuscript, the population of interest in your analysis are sexually active women. However, in the decomposition analysis, you used partner characteristics. This will restrict the analysis to married women or women currently in union. Are these two the same groups of women in Zambia? If not, please make sure that your analysis considered all sexually active women. Otherwise, you need to modify the title to fit to this consideration. | Thank you for this guidance. The analyis has been redone for Table 3. The unit of analysis is now restricted to sexually active women. All partner variables were removed from analysis. | **Table 3, pages 16-17** |
| 3.) In terms of relevance to family planning programs, traditional methods of contraception are of negligible importance. In the analysis, you considered folkloric and traditional methods in the same category as modern methods (see the dependent variable definition). Are these relevant in family planning programs in Zambia? | The traditional methods are not neccessarliy considered important in the family planning program in Zambia. However, it is note worthy to mention that there are still a proportion of women who are using these methods due to lack of access to modern methods expecially in hard to reach communities. |  |
| 4.) In the method section of the manuscript, you indicated that the women data were used for analysis. Yet, community level variables were also considered in the analysis. How were these community variables constructed or computed unless you used the household data? For instance, for community education, you need to produce the data from household data. Similarly, you need the household data for community wealth/affluence. Please provide a statement to clarify how you constructed these measurements in the method section. | Thank you for the guidance. A detailed write up has been inclided in the methods section explaining how community level variables were contructed using DHS data. | **Line 133-145** |
| 5.) How are the coefficient and endowment effects interpreted? You need to give your reader how the results are to be interpreted. | A write up has been included in the methods explaining how the coefficient and endowment effects are interpreted. | **Line 264-269** |
| 6.) In the conclusion part, you made the following statements:  'Changes in women’s contraceptive behaviour as a result of the continued FP promotion initiatives implemented over the 26-year period in Zambia, are responsible for the large share of the ongoing steady rise in the usage of contraceptives. Our study findings may reflect the need for further strengthening and up-scaling of family planning programmes in order to improve contraception services delivery, access and acceptance in Zambia in the quest to reduce high fertility and teenage pregnancy in the country.'  You have not included program related variables in the decomposition analysis. What finding led you to make this conclusion? | The statement in the conclusion has been edited. We await your further guidance. | **Line 414-417** |
| 7.) As a final note, the authors concluded that 'The study further suggests the need for increased targeted evidence-based interventions such as sustained investment for the education sector ...'. This recommendation is okay for future generation of girls/women joining the reproductive age. However, the finding also suggests that being uneducated serves as a barrier to contraceptive use. Thus, 'what might we do to remedy the challenges of the current generation of women that are less educated?' is also a pointer to potential recommendation for programs and policy. | Thank you for the guidance. We have added one recommendation based on your suggestion | **Line 420-423** |
| **Comments** | | |
| 1.) Providing a theoretical underpinning for research will help to inform your research methodology, including the variable selection and interpretation of your findings. By so doing, you can relate your results back to the existing theories and concepts, either by confirming or challenging them, and provide a deeper understanding of the phenomenon under investigation. | A theoretical framework has been included in the manuscript. | **Line 92-139** |
| 2.) Please go through the document and refine the language. For instance, on the method section of the abstract, please see below  'Adjusted coefficients with their 95% confidence intervals and percentages of were used to present results'.  In addition, in the Introduction of the manuscript  'Change in utilisation of contraceptive methods can could result from improvement in women’s social context such as improvement in education and women empowerment or contraceptive behaviour change.' | Edits have been to the sentence structure.  Edits have been made to the sentence structure | **Line 24**  **Line 83-85** |
| 3.) Please indicate the reference period for the decomposition analysis so that an appropriate interpretation can be made by your reader. | Thaank you for the guidance. This information has been inserted in the study title, objective in the introduction and methods section. | **Line 1**  **Line 88-89**  **Line 151-153** |
| **Reviewer 2** |  | |
| General comment: This study's writing and analysis are fine. But the tile is clumsy and needs revision. | We are grateful for your favourable comments and remarks. We would like to express our appreciation for your valuable expertise and the time you dedicated to our work. |  |
| Comment1. Based on the manuscript body, I would suggest rewriting the title as f: 1. Trend and socio-demographic factors in contraceptive use change among sexually active women in Zambia (1992-2018). A decomposition analysis OR 2. Social context in contraceptive use change among sexually active women in Zambia (1992-2018). A decomposition analysis | Thank you for the guidance. A title has been rewritten:  Social context in contraceptive use transition among sexually active women in Zambia (1992-2018): a decomposition analysis | **Line 1 on Page 1** |
| Comment 2: In the abstract section, the authors stated that: changes in women’s compositional characteristics such as education, fertility preference, number of living children, and experience of child mortality were associated with the increase in contraceptive use prevalence. This result should be supported by the valid statistic test formed to arrive at this result (Adjusted Odds ratio or p-value).” | Thank you for the comment. This has been effected in the abstract. The result is backed by the decomposition statistical test presented in Table 3 on page 15-16. The Blinder-Oaxaca decomposition analysis technique used does not produce odds ratio but produces coeffiencients with associated p-values and percentage contribution effect. Based on the results in table 3, we able to tell all the significant factors that contributed to the observed increase in contraceptive use based on the level of significant (p<0.05). The percentage contribtion shows. | **-Abstract on lines 30-33**  **-Table 3 on pages 16-17** |
| Comment 3: Discussion: I suggest adding one paragraph in the discussion section emphasizing the change in modern contraceptive use. That will contribute to the program improvement fusing on the modern contraceptive uptake in Zambia.. | Thamk you for the guidance. A paragraph has been included | **Line 310- 318** |
| Comment 4. Some references are not well presented and should be written as follows:  25. United Nations Zambia. United Nations Sustainable Development Cooperation Framework for the Republic of Zambia 2023 - 2027 [Internet]. Lusaka, Zambia; 2022 [cited 2023 Jul 20]. Available from: <https://unsdg.un.org/>  45. Ministry of Health. Reproductive Health Policy. Lusaka, Zambia: Ministry of Health; 2000 Jun.  Comment 5: The authors should add the link to access on the reference 45.  53. Hapompwe C. Sustainability of Universal Free Primary Education Policy in the Face of Declining Education Financing in Lusaka, Zambia. Afr J Educ Pract [Internet]. 2020 Jan 1 [cited 2023 Jun 29]; Available from: <https://www.academia.edu/43629975/Sustainability_of_Universal_Free_Primary_Education_Policy_in_the_Face_of_Declining_Education_Financing_In_Lusaka_Zambia>  54. Masaiti G. Education in Zambia at Fifty Years of Independence and Beyond: History, Current Status, and Contemporary Issues. 2019. | All references have been udapted accordinly. | **References on lines 521-647** |
| Conclusion: Well written with implications for program improvement of contraceptive use insight into the context in Zambia. | Thank you for the compliment. We appreciate your expertise. |  |
